# Discovering clinical phronesis

**DOI:** 10.1007/s11019-024-10198-8

**Published:** 2024-03-07

**Authors:** Donald Boudreau, Hubert Wykretowicz, Elizabeth Anne Kinsella, Abraham Fuks, Michael Saraga

**Affiliations:** 1 Faculty of Medicine and Health Sciences, Institute of Health Sciences Education, McGill University, 1110 Pine Avenue West, H3A 1A3 Montreal, Canada; 2 Centre Hospitalier Universitaire Vaudois, Av. de Beaumont 23, 1011 Lausanne, Switzerland; 3 Department of Medicine, McGill University, 3647 Peel Street, H3A 1X1 Montreal, Canada; 4 General Psychiatry, Centre Hospitalier Universitaire Vaudois, Route de Cery 60, 1008 Prilly Lausanne, Switzerland

**Keywords:** Phronesis, Clinical practice, Practical wisdom, Exemplary physicians, Qualitative research, Hermeneutic inquiry

## Abstract

Phronesis is often described as a ‘practical wisdom’ adapted to the matters of everyday human life. Phronesis enables one to judge what is at stake in a situation and what means are required to bring about a good outcome. In medicine, phronesis tends to be called upon to deal with ethical issues and to offer a critique of clinical practice as a straightforward instrumental application of scientific knowledge. There is, however, a paucity of empirical studies of phronesis, including in medicine. Using a hermeneutic and phenomenological approach, this inquiry explores how phronesis is manifest in the stories of clinical practice of eleven exemplary physicians. The findings highlight five overarching themes: ethos (or character) of the physician, clinical habitus revealed in physician know-how, encountering the patient with attentiveness, modes of reasoning amidst complexity, and embodied perceptions (such as intuitions or gut feeling). The findings open a discussion about the contingent nature of clinical situations, a hermeneutic mode of clinical thinking, tacit dimensions of being and doing in clinical practice, the centrality of caring relations with patients, and the elusive quality of some aspects of practice. This study deepens understandings of the nature of phronesis within clinical settings and proposes ‘Clinical phronesis’ as a descriptor for its appearance and role in the daily practice of (exemplary) physicians.

I have to make tough decisions with little information… it’s the nature of the beast.

A quote from a physician participant.

## Introduction

Phronesis is a concept that can be traced to Aristotle’s Nicomachean Ethics. In Aristotle’s view, it is a way of knowing and acting practically in the world. It is often described as a ‘practical wisdom’ adapted to the issues of daily human life. It is contrasted with abstract reasoning, such as pure mathematical (*episteme*) or philosophical (*sophia*) thinking. Phronesis relies on a capacity to navigate between general rules and the particulars of a specific situation. A person endowed with phronesis, the *phronimos*, is able to judge what is at stake in the situation, what means are required to bring about a good outcome and, indeed, what constitutes a good outcome. In the Nicomachean Ethics, phronesis is necessary to recognize and marshall a set of moral virtues, such as bravery, generosity, truthfulness or justice, relevant to a given situation. Moreover, phronesis is a ‘know-how’, i.e., the ability to execute the appropriate action, in an appropriate way, and at the opportune time. Fredrik Svenaeus described the concept as follows: “Phronesis, though not a moral virtue in itself in Aristotle’s philosophy (such as courage or temperance), is accordingly the ability to judge the right end of action in a particular situation and make a wise choice.” He concluded that “Practical wisdom and moral virtues are therefore mutually reinforcing traits.” For Aristotle, this know-how is grounded in the dispositions of the phronimos, nurtured and honed by experience “in concrete, practical matters of life” (Svenaeus 2022, 133–134).

Phronesis has been re-contextualized by numerous philosophers and social scientists, notably within a broader “turn to practice” (Bondi et al. 2011; Ellett 2012; Flyvbjerg 2001; Nicolini 2013). Hans-Georg Gadamer (1977) focused on phronesis as manifest through hermeneutic understanding and as a foundation of moral knowledge. Dunne (1993) contrasted phronesis with other modes of intellectual endeavors, notably *techne*, a reasoning aimed at a craft that results in the fabrication of an object (e.g., a house) or the production of a state of affairs (e.g., a safe journey). MacIntyre (1984) conceived of phronesis as enacted and revealed through specific practices. The conceptual landscape of phronesis has been explored in a variety of disciplinary fields (Kinsella and Pitman 2012), including education (Kristjánsson 2007), politics (Cameron 2018), law (Longan et al. 2020), and nursing (Jenkin et al. 2019; Flaming 2001).

Because phronesis guides decisions and behaviours in matters of human conduct and because medical practice requires careful and judicious conduct on the part of physicians, one would expect links to have been made between phronesis and clinical medicine. Phronesis in medicine tends to be invoked on the basis of two premises: that medical practice is a moral enterprise and that reasoning in clinical situations requires a distinct mode of rationality. Arguments based on the former line of logic have been developed and championed by Edmund Pellegrino and David Thomasma (1993). They view medicine as a unique human activity, the features of which are discoverable through its telos. In their opinion, the knowledge and personal attributes of the physician needed to fulfill the demands of that telos aggregate under phronesis. They consider phronesis the indispensible intellectual virtue for medical practice, one that integrates the moral virtues. Indeed, numerous authors have suggested that phronesis represents a paradigmatic approach to the understanding and teaching of clinical ethics (McGee 1996; Carnevale 2007). The rationale underlying this proposition may not be surprising given that Aristotle, himself the son of a physician, used the medical analogy extensively in developing his theory of ethics as a practical science (Jaeger 1957). A consequence of foregrounding ethical deliberation is that the body of empirical literature on phronesis in medicine, which is relatively small, reflects a predominance of analytic studies focused on ethical dilemmas (Paes et al. 2019; Torjuul et al. 2005; Kotzee et al. 2017; Jameel 2022).

A second justification for exploring the role of phronesis in medicine is epistemological and is grounded in hermeneutic explorations of the illness experience, the clinical encounter, and clinical understanding. If one considers medical rationality as interpretive, then the pertinence of a practical rationality, one that incorporates moral common-sense, becomes obvious. This has been advanced by Gatens-Robinson (1986) and Widdershoven-Heerding (1987) and further elaborated by Montgomery (2006), Kaldjian (2014), and Svenaeus (2022). Pellegrino and Thomasma (1993) described how clinical judgement requires practical wisdom. Kaldjian extended that supposition by suggesting that clinical judgement is *itself* constituted by practical wisdom. Svenaeus (2022) explored the nature of good medical practice, focusing on differences between understanding and explanation. The former is described as follows: “To understand in medicine from the doctor’s point of view implies to be understand*ing*, which means attempting to put oneself in the patient’s situation. This bridging takes place through empathy, guiding and assisting clinical understanding, but the empathy does not exclude a critical, productive distance, since the doctor’s understanding belongs to a professional horizon manifesting a specific kind of medical interpretation” (Svenaeus 2022, 160).

The body of literature on phronesis is overwhelmingly theoretical in nature. Phronesis, as enacted in medical practice (and, for that matter, in other domains) has only recently been examined on the basis of empirical data and as noted above, primarily with a focus on moral adjudication. We cite four research programs that have resonance with the aims of our inquiry. A cross sectional study by Jameel (2022) explored how phronesis was manifested in the family medicine practices of 16 exemplary physicians. Jameel conceived of phronesis as a constellation of 34 constitutive elements, using a school of fish as a visual metaphor to represent the concept. The result is intricate and somewhat abstract. Another exploration of practical wisdom was undertaken by a research group at the University of Birmingham. The motivations, rationales, findings, and outcomes of this multi-phased program are summarized in a final report (Conroy et al. 2018). This work was part of a broader program of research (Kotzee et al. 2017; Malik et al. 2020) focused on a ‘deliberative’ interpretation of phronesis, namely the structure of ethically wise decision making. The research was conducted with a deductive approach to analysis, based on the ‘goals of medicine and goals of care’ framework proposed by Kaldjian (2010). A third study, this time with a social constructivist frame and an inductive approach, with similar goals of understanding decision making in the face of ethical dilemmas, was conducted by Paes and colleagues (2019). A fourth paper, recently published by Lauris Kaldjian and colleagues (2023), investigated practical wisdom in medicine as understood by medical students and physicians. Using a highly structured study design, incorporating both inductive and deductive approaches, they found that participants conceived of phronesis as a virtue that is “deliberative, goal-directed, context-sensitive, integrated with ethics and marked by integrity and the motivation to act.”

The research we present below differs from these previous inquiries in two respects. First, we privileged in-depth interviews with an iterative inductive approach to analysis. Second, we focused on physicians’ usual practices rather than decision making confined to ethically challenging clinical situations.

We set out to explore the nature of phronesis in quotidian clinical practice. We did so by examining empirical material gathered from exemplary and experienced clinicians who discussed their work in interviews intended to understand their medical work. We searched for instances of phronesis in order to describe its dimensions and importance in clinical medicine.

## Methodology

This paper stems from a re-analysis of a series of eleven sets of transcripts of semi-structured interviews of physicians, respected by their peers for their clinical excellence, and involved in teaching medical students and residents (Saraga et al. 2019). The participants included four women and seven men, with a range of 13 to 40 years of post-residency clinical experience, from the specialties of general surgery, pediatric surgery, emergency medicine, family medicine, cardiology, nephrology, obstetrics-gynecology, general internal medicine, and psychiatry. The initial study used interpretive phenomenological analysis and found that clinical practice as a lived experience can be broadly described as ‘engagement’ in the clinical situation. Furthermore, we noted that the findings pointed to phronesis as a central dimension of the practice of these exemplary physicians. Therefore, the research team undertook a secondary analysis of the data, this time focused on phronesis. The aim was to elucidate the nature of clinical phronesis and its place in medical practice. We used a hermeneutic and phenomenological framework for the secondary analysis (Gadamer 1977, 1996a, 1996b; Heiddeger 1962; Jardine 1992; Kinsella 2006; Moules et al. 2015; Ricoeur 1991).

Our approach is phenomenological and oriented toward a rich description of the human lived experience we wish to elucidate, in this case, the lifeworld of medical practice, gleaned from the points of view of the physicians’ verbal renditions. It is also hermeneutical because, as Ricoeur suggests, accessing the meaning of lived experience requires *mediation*. For Ricoeur, mediation requires interpretation of works of culture; in our case, these works of culture are the physicians’ own narratives. In most instances, the transcripts disclose actual encounters and verbal engagements with patients while others are stories of physicians’ actions on behalf of patients under their direct care.

The hermeneutical approach (Gadamer 1977, 1996a; Ricoeur 1991) assumes working within an epistemological circle, that is, starting with a pre-understanding of what is at stake and putting it to work when confronting the research material. At the same time, avoiding a self-fulfilling outcome requires at least two steps. First, it involves an explicit starting point *in the tradition* (Gadamer 1977); in our case, in the works of Aristotle and Aristotelean scholars. From a hermeneutical perspective, this pre-understanding is not a bias, but rather a means to explore the narratives of the physicians. Second, it depends on attending to the ‘things themselves’, listening to what the physicians themselves say, instead of discovering what we may already know.

We consider the concept of hermeneutics in three different though related senses. The first is the epistemological meaning of an interpretive analysis of a text — in our case, the transcripts of the interviews that constitute our primary research data. The second refers to an understanding of clinical medicine as a form of dialogical hermeneutic practice. We were conscious that the testimonies of some of our physician participants, when they gave accounts of patient’s words, signs, and behaviours, could be construed as, “meeting between two persons [physician and patient] and an interpretation of the ill person’s being-in-the-world, with the aim of restoring a life that has turned unhomelike” (Svenaeus 2022, 91). Third, we turn to a deeper meaning of hermeneutics, as described by Heidegger and Gadamer. Their ontological framing of hermeneutics provides an entry for considering the being-in-the-world of physicians. What is underlined is the nature of the “world”, that is, the clinician’s world, comprised of clinical situations. Clinical practice is a “being-in-the-clinical-situation”, of which the patient is a focal point, but whose horizon encompasses much more. In other words, the object of medicine as a hermeneutic endeavor encompasses the whole of the clinical situation. While there is substantial literature on the lifeworld of patients, there is much less on the lifeworld of physician practitioners — the focus of our research.

We bring pre-understandings and situated experiences as academics, theorists, and practitioners with a scholarly interest in phronesis, into conversation with physicians’ accounts of their clinical practices. The research team brought disciplinary perspectives from medical education, internal medicine, psychiatry, philosophy, philosophy of education, and philosophy of medicine. Our team has scholarly expertise with phronesis, having published manuscripts, book chapters, and books focused on various aspects of phronesis and professional practice (Boudreau et al. 2018); (Boudreau and Cassell 2021); (Fuks et al. 2012); (Kinsella and Pitman 2012); (Saraga 2019); (Wykretowicz  2011).

At the outset, the research team met on three occasions to discuss pre-understandings and interpretations of phronesis. Our work began with conversations about the scholarly literature on phronesis and readings from influential thinkers. The team members discussed how they ‘came to’ phronesis in their scholarly work. We then engaged in dialogue regarding the analytic focus and the research question of the inquiry. After several iterations, the following question emerged: *How is ‘phronesis’ manifest in exemplary physicians’ stories of clinical practice?*

The team met every 3 to 4 weeks over the course of a year to discuss the transcripts; each team member read all transcripts in depth. Throughout the process, researchers created mind maps of key aspects of phronesis revealed in the texts, noted excerpts from the transcripts that ‘showed’ phronesis, dissected clinical stories that revealed phronesis, and reflected on what ‘hit a chord’ during engagement with and close reading of the texts. The initial notes from the mind maps of each team member were collated, and reviewed iteratively, by two subgroups, and then collectively by the whole team.

The overarching themes we identified were: ethos, clinical habitus, encountering the patient, reasoning amidst complexity, and embodied perceptions. Each of the transcripts was revisited and coded for these themes by a research assistant. The coded data were extracted from each transcript according to each theme. A data set of 12–20 pages for each of the five themes was compiled from the coded data. This served as the basis for the verbatim citations that are presented in the findings section of this manuscript.

Our study received continuing review and approval from the Institutional Review Board of the Faculty of Medicine and Health Sciences of McGill University on April 18, 2021 under the IRB Study Number A04-E18-21 A (21-04-045).

## Findings

Below we present the themes drawing on descriptions of phronesis identified through sustained deep engagement with the physicians’ accounts of practice. Our aim is to ‘show’ the data, rather than to ‘tell’ what was found, so that readers can bring their own interpretations to the extracts from the transcripts and see the basis of our interpretation. As is common in qualitative research of this nature we present the thick descriptions from the raw data in this section and follow with a detailed analysis in the discussion. In addition, at the end of each of the thematic subsections we provide a link between our empirical findings and philosophical concepts, notably hermeneutics and phenomenology. Finally, while the themes are presented separately, it is important to note that this is an instrumental disentanglement, as much of the data could be placed in more than one thematic category. The dimensions of phronesis that we discerned in our analysis of practitioners’ accounts of their clinical practice are inevitably intertwined.

### Ethos

The physicians offered detailed descriptions of clinical practice that revealed aspects of character, that is, what they value, who they are as persons, and who they have become as physicians — their ethos.

The sources shaping character were a recurrent topic of conversation. Early experiences and role models, both parents and clinical teachers, were described. A psychiatrist stated:

People who are good clinicians – that resonated with me. And what went into my development so that the role models that I had were able to impact on me. You know it probably had as much to do with my family and my parents as it had to with my role model physicians. (P-2).^1^

The same participant recalled how, when he was a teenager, his physician was “a warm compassionate human being” and how that was formative (P-2). Another participant discussed how lived experiences become “embedded… in your character, in your being” (FU P-4). Similarly, a family physician stated, “Why did I end up the way I am? I guess I blame my family, my parents, my upbringing” (P-8). And, an emergency room physician reflected on experience and character:

I believe this comes, again, from a cumulative experience of how you were taught to see the world by – as a child…the people you’ve met over the course of your lifetime who have influenced you, the books you’ve read, you know, your humanity, your interest in other people. (FU P-9).

Participants underscored moral dispositions as important to good practice. For instance, clinical humility, along with qualities such as “understanding your own limitations,” “not being so egotistical,” and “always trying to learn” (P-1). One physician referred to himself as “an everyman” articulating his ethos to “work hard” and take his job “seriously” (FU P-2). Humility was also linked to truthfulness. A pediatric surgeon explained, “by sense of humility, I mean to admit your mistakes, to admit that things could have been done better” (P-4). An internist highlighted humility as linked to “an honest practice” stating “if I make a mistake or an error I will admit it; if I forget to do something, I will tell them I forgot,” and reflecting further, “and if I don’t do it, I feel bad about myself…I hear my mother’s voice in my head and things like that” (P-10). For some, humility was linked to “putting the needs of the system before yours” and to “accountability” (P-7). This was echoed by a surgeon who described a “personal sense of accountability to people.” With respect to its origin, she added that it “was something I learned very early on” (P-1).

A commonly expressed element of character was a capacity for hope and optimism, which at times edged into faith. An emergency room physician shared that “you want to always have people around you that have hope. And in most cases, hope is appropriate. It’s a rare case where there is no hope” (P-9). A surgeon, dealing with a difficult operation, explained that “it was really an act of faith. You know, just hoping and praying that I am wrong and that, despite all my medical knowledge, that this will still turn around” (P-4). Others invoked the metaphor of a glass half-full: “I love what I do; I’m enthusiastic…I think I’m a positive person” (P-6); “if you want to look at that [glass] half empty, then you look about all the things that haven’t worked and how frustrating it is, but that’s not my personality” (FU P-7); and “People say you’re always happy, you are always smiling, you always put a positive spin on everything. I really love what I do, and I try to model that behaviour for other people” (P-8).

The disposition of being available and responsive was common:

If they ever called me with a problem, I was quick to respond and maybe did a good job. …My colleagues still, if they have a family member, a patient in distress, I will respond the same day. (P-7).

Seeing one’s work as a vocation rather than a job and wanting to make a difference was noted frequently:

These kinds of people who just see it more as a vocation than a job, which I think is what it should be, frankly, given what we deal with on a day-to-day basis…in the vocation, there’s a certain passion and intensity that allows you to extend beyond the rules. (P-7).

A surgeon described this disposition as “a desire to be significant” (FU P-4) and an emergency medicine doctor as “I have the opportunity to make a difference for many more people and that’s personally rewarding for me” (P-9).

Several participants referred to conscientiousness and attention to details, at times bordering on compulsiveness. A nephrologist linked this characteristic to a “fear of missing something and doing something wrong” (P-3). A general internist noted, “I guess I’m sort of a tidy person…It means I’ve checked off my list of things to be done” (P-11), and “I’m kind of a systematic and organized person…that sort of helps me in taking care of patients” (FU P-11). The conclusion of this physician was, “that’s just part of the way I was born” (P-11).

That there is a set of personal characteristics deemed to be suited to the practice of medicine is hardly a novel proposition. Innumerable commentaries are premised on the idea that moral dispositions are at the heart of medicine (Bryan 2009; Pellegrino 1993; Walker 2005). It is well aligned with classical Aristotelian views of moral virtues as grounded in the individual’s character — *moralis* in Latin or *ethos* in Greek (MacIntyre 1984, 38). In contrast, and regrettably, the notion of character has been supplanted by related notions such as personality, professional duties, or the unfortunate and incongruous entity ‘behavioural competencies’. There is, however, general agreement on the desired personal attributes or ‘excellences’ fitted to doctoring (Boudreau et al. 2018). Our physician participants make mention of compassion, humility, truthfulness, and conscientiousness — these often appear on lists of characteristics of the ‘good doctor’. They also insisted on a capacity for hope; it is an aspect of the medical ethos that is less salient in the academic literature, though perhaps not in the lay press.

### Clinical habitus

Our participants described particular ways of how they go about getting things done in their clinical practice.

A general internist underlined the importance of not interrupting the patient prematurely: “Every visit, I try to give [patients] a little forum at the beginning to make sure I know what they want to talk about and try not to interrupt too soon… I make a conscious effort to do that” (P-10). This ‘know-how’ was linked to the notion of holding off on judgements: “You are there not to judge them…you are there to help them with their health issues…I want people to trust me” (P-10). A psychiatrist expressed something similar: “in establishing trust with patients…I take a non-judgmental stance…I’m understanding it without judging it” (P-7).

A family doctor conceived of his clinical work as one of offering guidance: “My job is to be more of a guide and a resource…to be there for them if they need me, to help them and give them some direction, but not necessarily tell them what to do” (P-8). Another participant expressed what it means to ‘be there’ for patients as follows: “I’ll be there for them…if someone is not happy about something then I’d like to fix it. You know if someone feels like they’re in crisis, we’re there to help [them] through it” (P-3).

Helping patients getting through clinical visits at times required forethought and meticulous groundwork. As an illustration, a nephrologist, anticipating a need for dialysis in the future, introduced that possibility gradually over many prior months so that her patients “are ready by the time they need to do this treatment.” She added that “the manner in which it’s accepted by the person is part of your treatment, you know” (FU P-3). This physician explains to patients, “we need to prepare you…and we’re gonna do things a little bit at a time” (FU P-3).

Another ‘trick of the trade’ was revealed through the complex maneuvers sometimes necessary to make patient visits possible. “I don’t think he ever realized to what extent I had squeezed him into clinics that had no space, saw him on days that I had no clinic… and if he didn’t show up to an appointment, remade the appointment for him” (P-3). These kinds of adaptations were described as a response to a system that may seem broken, “things that we try to do to accommodate for a system that doesn’t change, for things that get lost” (P-3).

Knowing how to do things may involve setting the tone for the clinical environment, as was vividly described by a surgeon talking about the operating room and his role as a “tone-setter”:

There are times in the operating room where a fatality feeling starts to set in – that we’re going to lose this patient, and it becomes a self-fulfilling prophecy…I often find myself the re-setter for peoples’ tone… Let’s focus on this…that’s one of the things I think I do well. I don’t get all anxious and start just sort of panicking…once a tone of panic sets in, it’s amazing how infectious it becomes…the next thing you know…the whole team is no longer communicating; everybody’s just in panic mode. (P-4).

One participant discussed clinical know-how as “street-smarts”: “that’s streets-marts, right – someone who knows – who recognizes something bad when it’s bad; something good when it’s good; how to get out of trouble; how to not get into trouble, you know. This exists in medicine” (P-9).

These dispositions, which we have designated clinical habitus, are a facet of practical wisdom. The findings underscore the importance of experience in their development. The cumulative experience shapes a set of dispositions of a pragmatic nature that allow the physician to be “completely geared to the demands of the situation” (Dreyfus 2014, 81) This is a major aspect of the practical wisdom which consists of “the process of bringing a thing or situation from unintelligibility to understanding” (Svenaeus 2022, 107). A focus on clinical habitus reminds us that “medical practice is to be understood as a special form of understanding, which is identical with neither explanation in science nor interpretation in the humanities” (Svenaeus 2022, 108).

### Encountering the patient

A theme, overlapping with ethos and habitus and with striking salience in our data, was the attentiveness of physicians to the unique needs and situations of individual patients. Participants frequently viewed patients as family and cared for patients as they would wish a member of their own family to be treated. As one surgeon explained, “there’s no checkbox saying, ‘would you let this person operate on your daughter?’ which is the bottom line, because this is what the patients want to know when they come in at the end of the day” (P-1). She elaborated, “I think that is often the thing that runs through my head, ‘if it was my mother, father, husband I would want the best person possible for them’” (P-1). “I approach patients as if they’re family members, so…if I operated on them and they’re in the hospital, I’m not going to let someone else look after them while they are in the hospital; it would be me that sees them” (P-1).

The patient’s perspective and experience are at the centre of care. One physician stated, it is “the patient who has the illness; you know, the person – the persons’ history, and their perspective and their experience, their subjectivity.” He went on to discuss a clinical situation where this “was real; it wasn’t like lip service” and where patient experience was built into “the fabric of what we did” (FU P-2).

A cocooning aspect to the clinical dyad was frequently noted, described as “being-in-the-moment” or offering “undivided attention.” One participant used the metaphor of “being in a bubble,” to describe a role-model physician who “was able to focus – have the patient focus on him, on the process…just that attentive, active listening and questioning in a busy place, but they somehow…seemed like they were alone” (FU P-10). This “implies that their attention is dedicated to the person in front of them…they are able to clear their mind and their duties for that brief period…able to focus” (P-10). He noted this bubble of undivided attention could occur “anywhere.” “They were able to do it anywhere. Bedside, stretcher in the hallway…it’s not the most private place in a busy emergency hallway but they were able to do it there, at the bedside, in a lounge chair in the solarium or whatever” (P-10). An emergency physician described something similar:

You have to make sure that the patient gets the feeling that you are in that minute…you have to give the impression that [you are] in the moment……I don’t mean just look them in the eye……but I mean absorb the moment that you’re with them. (P-9 and FU P-9).

Several physicians talked about “giving their all” to patients. A cardiologist stated: “I’m your doctor, you put your trust, your faith in me, and I’m gonna do everything I can for you, no matter who you are”. He went on to say, “I try to give my 100%, and no matter if it’s a follow up for ten minutes, or if it’s a new consultation, or if it’s someone – a VIP or whatever – I treat everybody the same…so it’s not very complicated, and I just give my all” (P-6).

Relatedly, the physicians’ stories offered illustrations of respectful behaviour, such as self-introduction and referring to the patient by name rather than disease category or hospital room number:

I made sure that every room we go in, I introduce myself and the student and what he is here for…no matter how busy you are, you need to introduce yourself to the patient and tell them who is in the room…I mean, these are all very basic things, but often they’re left undone. (P-4).

The imperative of listening to patients was a prominent topic of discussion. One physician estimated that 75% of patients were helped “just by listening to them”. He stated, “you don’t actually offer any solutions. You just listen and they say ‘thank you’ (laughs) and they go away happy. I used to be a bit puzzled and…I didn’t solve their problem…it might have just been that someone actually listened” (FU P-11). “…the effort is listening – I think it’s the active listening side and finding something to latch onto in the patient’s story… you do have to make the initial effort” (FU P-10). “…the things that I think I attach particular importance to are that I listen to the patients that are there. I respect them, respect what they have to say and the manner in which they would like to be treated” (P-3). And, “[what] I strive to do is to be respectful to the patients, to listen to them, to treat them as human beings, to be a nice person, to make them feel comfortable about coming to the doctor” (FU P-6). The act of listening was of pragmatic value: “number one reason I made that diagnosis is because I listened” (P-9). Furthermore, “in that clinical bubble, you’re trying to get at an underlying disease…[a] goal that’s very clear – and listening for clues…is obviously the ultimate goal…” (FU P-10).

One participant summed up his views quite simply as: “When you’re one-on-one with your patient, you should give them the time; listen to them; be careful not to hurt them, not to harm them; make sure you don’t make mistakes. If you make a mistake, you tell the patient, ‘I’m sorry I made a mistake,’ and discuss it with them” (FU P-6).

The importance of the encounter between physician and patient is woven throughout the interview material and represents a major feature of medicine as a hermeneutic endeavor. Marianne Paget, in a phenomenological interpretation of clinical work, describes it as: “a practice of responding to the experience of illness. As such, its context is a relational encounter between persons about the afflictions of the human body and the human spirit. It is grounded here in this relational encounter from which it typically departs and to which it typically returns” (Paget 1988, 21).

### Reasoning amidst complexity

All participants reflected on the complexity of thinking and decision making amidst the uncertainties and contingencies inherent in the practice of medicine. For instance, an emergency physician stated: “I have to make tough decisions with little information with severe consequences, but you have to make the decision…it’s the nature of the beast” (FU P-9). A pediatric surgeon discussed the unpredictability of clinical contexts by highlighting the absence of fixed and immutable anchors: “one thing I know in medicine is that there is no zero and there is no hundred – those numbers don’t exist” (P-4). Another surgeon alluded to the “betwixt &amp; between” feel of practice, where clinical goals are subject to change: “‘Get better’ is a moving target… and if we teach our older students [that] you’re trying to restore health, it doesn’t necessarily mean they [the patients] don’t have an illness” (P-1).

A psychiatrist noted that his clinical reasoning was influenced by a recognition of the limitless range of patient characteristics and the incompleteness of what can be known about patients: “You know people are complicated. There’s lots going on. We see little bits of it. We need to know that we’re only seeing little bits and understanding little bits and try to understand more” (P-2).

Another psychiatrist described taking various contingencies into account in her deliberations on prioritizing admissions: “I have almost a dilemma between the patient in front of me and the general picture of all the people waiting for care” (P-7). She described weighing “pertinent factors” to guide her actions:

We kinda feel – a young person who’s never been hospitalized, where someone who has been hospitalized ten times – if they’re equal, then the person who’s never been hospitalized should have a chance. That kind of decision, you know – ethical like. There are issues like school. If they don’t get in, in June, then they won’t get out to go back to school; so that’s a factor that gets entered in. (P-7).

Those two quotes, by psychiatrists, illustrate how clinical thinking involved polarities. They refer to ‘lots’ vs. ‘little bits’ and the ‘general picture’ vs. ‘pertinent factors.’ There were multiple expressions of that nature in the accounts. A pediatric surgeon stated: “I think it is part of our training that we don’t just think in one arena; we look at the global picture…Whereas, people who are just focused on their one task, they just stay on their one task until they succeed or fail” (P-4).

One participant discussed how she holds the big picture of a patient’s prognosis while attending to current issues:

I always have kind of a big picture whenever I see somebody – I have sort of an idea of what the next weeks will be like, and what the next months will be like, and what the next years will be like…then I kind of whittle it down into little parts of digestible segments. (P-3).

An emergency room physician described how a student reasoned toward a faulty diagnosis by attributing too much weight to a few symptoms, thereby missing the forest for the trees:

I was talking with a medical student yesterday how in the case she saw with me – someone who was anxious, shortness of breath and palpitations – and she thought for sure she had a pulmonary embolism because these are three symptoms you can get, when it was completely evident to me that she was just very anxious. And I spoke to her that she’s seeing the individual trees but not the big forest that made up a person, that made up someone that is anxious. (P-9).

One participant highlighted the risk of drowning in details, and how wise physicians mitigate information overload by foregrounding the most salient aspects of a situation:

Sometimes patients are…very complex. They have fifteen different issues going on and you might get put on the list, and sometimes people just drown in the details – and then some people have the ability to say, ‘Okay, this fits with this, and this doesn’t fit with this, and this is the most important thing, and this is the second thing’, so they can take all this morass of detail and structure it in a way that makes the patient manageable. (P-11).

The choreography involved in thinking about the general aspects and the particularities of a case came to the fore when clinicians discussed their use of algorithms. For example, a cardiologist, giving an account of his reasoning about a patient suffering from coronary artery disease, stated:

He’s diabetic; he’s got three-vessel coronary disease. According to the guidelines, this patient should go for surgery. But, having seen everything that happened with other surgeries – he’s infected everything that’s ever been put in – and with this whole prosthesis and all that stuff, the thought of this guy having a bypass operation and somehow getting through it without being put on long-term dialysis therapy…I basically discussed with him and I said, ‘Listen, I could send you to surgery. My feeling is that you wouldn’t be better after it.’ (P-6).

Clinical practice often proceeds in the midst of uncertainty, complexity, epistemological confusion, and emotional upheaval. The cardiologist’s use of the phrase “my feeling” is revealing, undergirds the idea that clinical rationality is more than ratiocination, is highly complex, and difficult to characterize. Kathryn Montgomery has described medicine as “an acquired rationality — culturally engendered, communally reinforced, interpretive, situationally sensitive and therefore dialogic and aphoristic in character” (Montgomery 2006, 165). Clinical judgement may be conceived as grounded in an experienced knowing and understanding; as Gadamer contends, it is precisely an intelligence of the situation that in turn leads to wise judgement (Gadamer 1991, 36). Gadamer’s assertion that “the Aristotelian virtue of wisdom—phronesis—is the basic hermeneutic virtue itself” (Svenaeus 2022, p 132) could readily be applied to our understanding of the nature of physicians’ reasoning amidst the complexity of medical practice.

### Embodied perceptions

As highlighted in the last quote, certain clinical decisions can be based on embodied perceptions, sometimes described as “gut feelings”. A cardiologist explained: “following these gut feelings where you just feel it’s the right way…like you’re being pushed in a certain way, that you just need to kind of be like a leaf in the wind, and just…go where you’re being kind of guided” (FU P-6). A surgeon noted: “some of your movements come from a gut feeling that is the right thing to do; some of your movements do come from experience that ‘I’ve done this before – I’ve had a patient bleeding from the spleen or bleeding from a large blood vessel before’” (P-4). He described how his “gut feeling” shaped a particular case:

I did what my experience tells me to do, which is pack that pack…yes, this is the way we do it…but in this particular case, we need to do [something else], we packed just enough to let the sponges absorb the blood…to get as much of the blood out, [then] took that out and found the vessel…and that actually worked. (P-4).

Several participants referred to ‘instincts’ or ‘intuitions.’ A psychiatrist stated: *“*I go up to see her during rounds, and I have… you know the instinct of a physician where you think the patient’s gonna die any second?” (P-7). Similarly, in describing a 57-year old man, a family physician explained:

[He] met the criteria for major depressive episode [but there was] no past psychiatric history, no family history…no alcoholism…it didn’t fit my typical pattern…When I went through it in my mind… there was just something inside of me that said…I call it intuition, I said deep down, there’s something wrong with this story. (P-8).

Such a feeling can be elusive: “Sometimes …you just get worried…he didn’t look really well…And it’s hard to actually put a finger on exactly what it is, but it’s quite striking” (P-11).

Some participants referred to intuitive clinical impressions, for instance as being able to see the shape of a patient’s illness or prognosis, almost as a ‘gestalt’:

I looked at her…she had a massive pulmonary embolism, and like I just saw it in her, right away. I’ve seen that in other cases…I’m seeing the disease as a physical manifestation…I felt so much like this guy who says he didn’t see the numbers; he saw a shape – this was a shape I was seeing that was the shape of pulmonary embolism. (FU P-9).

Impressions of this nature were sometimes at odds with standardized guidelines:

There are patients that may be along the algorithm now where surgery would be recommended, that I don’t send for surgery…because I’ve got this feeling that they’re gonna be dead – they’re not gonna make it through …on paper, you’d think that that was the right thing to do, but it’s like somehow…I just feel that they’re not gonna make it. (P-6).

One participant commented on these types of impressions as related to a certain clinical sense, noting that it may be absent for some physicians:

You know, you can look at diagnostic studies…there’s still a certain clinical sense that will never be really quantified…there are some people…who don’t have any clinical sense at all, who purely go by objective data; and there are people…who just come close to a patient’s bedside, look in their eyes, and say, ‘I think they’ve got this’ (laughs). (FU P-4).

A number of participants described complex and ineffable situations where, in addition to relying on gut feelings, instincts, or intuitions, they enlisted prayers when considering what may be a desirable clinical course of action: “It’s when we go off the algorithms that it’s a little bit more complex…and we have to rely on our better clinical judgement or what we think is right, and you hope is right, and sometimes it’s a little bit of prayers involved” (P-6).

The recurrent comments regarding clinical sense and a need to reconsider algorithms speaks to a form of attunement of an experienced physician to the large array of elements in the clinical situation, some of them not readily reducible to objective data. As we noted before, when speaking of the clinical habitus, clinical practice is a hermeneutic, endeavour which is so deeply embodied that its mastery is more of a “skillful coping” than a rule-governed judgement. Dreyfus illustrates this as follows: “when I enter a room I normally cope with whatever is there. What enables me to do this is not a set of beliefs about rooms, nor a rule for dealing with rooms in general and what they contain; it is a sense of how rooms normally behave, a skill for dealing with them, that I have developed by crawling and walking around many rooms” (Dreyfus 2014, 89). These observations are not tantamount to considering scientific explanatory reasoning as irrelevant but rather to realizing that practical wisdom goes beyond scientific reasoning. As Ricoeur would suggest, science can explain more so as to understand better (Turoldo 2018).

## Discussion

Contemporary discourse on phronesis in the health professions has generally been tethered to its philosophical underpinnings in the Aristotelian tradition and centred on ethical decision-making. There have been few empirical explorations of phronesis and its enactment in the actual lifeworld of medical practice. Our study’s intention was to reveal and understand how phronesis is manifest in the experiential accounts of expert physicians. Our analysis brought to light the following elements of phronesis: ethos, clinical habitus, encountering the patient, reasoning amidst complexity, and embodied perceptions.

In this section we engage in further reflection on our findings in order to elucidate the phenomenology of clinical practice. Our study is based on the idea that a hermeneutic phenomenological lens to examine phronesis in practice can contribute to a richer understanding of the being-in the-world of physicians. Indeed, their accounts revealed the nature of the challenges and predicaments they face — in particular the complexity and contingency of clinical work — and how a prudent *savoir faire* helps physicians fulfill their duties and responsibilities.

Our discussion highlights how the initial theoretical framing of classical phronesis places the empirical findings into relief, namely: the contingent nature of the clinical situation, a hermeneutic perspective on clinical thinking, the tacit dimensions of practice as revealed through practical know-how, the centrality of caring clinical responses, and a certain “je-ne-sais-quoi” or intuitive and less tangible quality of practice. Each of these are discussed below, linking back to the theoretical foundation and supported by numerous citations to the empirical data.

### The contingent nature of the clinical situation

The clinical situations described by our participants are characterized as complex. Complex situations in the data were linked to “little information” (P-9), the “morass of details” (P-11), “moving targets” (P-1), “complicated people” (P-10), “dilemmas” (P-2), and the “many arenas” (P-4) pointing to the contingent nature of clinical practice. In Aristotelian terms, this “contingent world” represents that part of reality that “could be otherwise” (Aristotle 1999, 89 [1140a]). Phronesis is the mode of thinking and acting with regard to the contingent world. Indeed, a central feature of phronesis for Aristotle is that it is oriented toward decisions and actions. This contrasts with the “necessary world,” i.e., the part of reality that must, of necessity, be as it is, therefore not requiring deliberation. Practical understanding and action take place in the contingent world, whereas theoretical thinking deals with universal and necessary rules, for example, mathematical propositions. Since, in our view, clinical practice is made up of a series of clinical situations, it is important to clarify what we mean by a ‘situation.’

Our data align with a phenomenological view that situations have three important characteristics (Heiddeger 1962; Sartre 2003). First, a situation is always more than an example of a generic category; rather, it is a singular set of contingent elements facing an individual. For example, the situation of the diabetic patient with three-vessel coronary disease extends well beyond coronary morphology and guidelines on how to manage similar cases. Second, the situation is not given as such to the clinician; rather, the clinician helps shape and sharpen a reality that is blurred at first, transforming it into a meaningful and actionable situation, what one participant alluded to as a “big picture….an idea of what the next weeks (and) months will be like” (P-3). Thus, the situation is not an objective given, a picture passively laid out in front. We can see it emerge from the dynamic interplay between the clinician and the environment and its many actors. The situation is construed as a field of affordances and hindrances; it triggers the clinician’s predispositions, disclosing possibilities, and requires an understanding of what is at stake as well as a capacity for taking appropriate actions (Gibson 1986; Noë 2004). Third, clinicians envisage situations as a complex web of heterogeneous and intertwined concerns, not limited to strictly biomedical issues, but including the broader medical and social issues at stake: the patient, most certainly, but also the family, the nurse on call, the overworked radiologist, the recalcitrant spleen, the new practice guidelines, the crowd in the waiting room, the pressure of time, etc. These varied and relevant aspects of medical practice have been described in a variety of contexts. This perspective corresponds with an understanding of practice as described by Joseph Dunne; in contrasting the rationality of practice with technical rationality he points out that the latter pays insufficient attention to the “socio-political, institutional, and historical matricies within which individuals themselves are located” (Dunne 2005, 382). Similarly, Svenaeus noted that “Phronesis is not devoid of feelings, it is rather based in feelings that help the morally wise person to see and judge what is at stake in the situation” (Svenaeus 2022, 135). In sum, a situation is at the same time: (1) contingent (Aristotle) (2) emergent from a dynamic engagement of the clinician with the total clinical environment (Sartre 2003) and, (3) heterogeneous and complex in nature (Ingold 2021). Thus, it is not surprising that clinical phronesis was revealed when physicians confronted and responded to murky and challenging clinical situations.

### A hermeneutic perspective on clinical thinking

Our results indicate that the clinical thinking of the phronimos is distinct from a traditional view of rationality, one that subsumes the particular case under general laws. To go back to the example of the diabetic patient, the cardiologist does not simply categorize the patient as “having a three-vessel disease” (P-6) and then applies the guideline of recommending surgery. Rather, what this participant describes is a hermeneutic engagement in the situation. Clinicians must gain an understanding of what is to be done through a number of interpretations, whose objects include inter alia: the patients, their past history, their environment, the clinicians’ environment, and the guidelines themselves. Such a perspective raises a number of issues. For example, there are limitations and constraints. Clinicians’ understandings are always partial and there is a risk of missing something important. As one participant said, “we only see little bits” (P-2). It is the case that clinicians are never able to obtain a God’s eye view—what Nagel (1986) refers to as a “view from nowhere”—even though anonymous algorithms might appear to provide complete clarity. Therefore, interpretive, albeit constrained, engagement is the only way to gain an understanding of what is at stake. In this view, subjectivity is not a ‘bias’ that should be corrected, as a strict cognitivist would have physicians do (Kahneman et al. 2021). Our findings suggest that the physicians’ interpretive engagement in the multidimensional clinical situation allows them to make sense of a messy reality: “they can take this morass of detail and structure it in a way that makes the patient manageable” (P-11).

Clinical thinking is not simply rational decision-making but rather *a mode of orienting oneself* in the practical world, such as resetting the “tone” of the operating room and getting everyone to focus on the task at hand. This involves a hermeneutic back and forth between the general and the particular. In our data, indeed, we find many different kinds of ‘generals’ and ‘particulars’: patients and their illnesses, the body and its parts, foreground and background, big picture and focal point, long-term and short-term, principles of medicine and individual cases, guidelines and the demands of the situation, best practices and systemic constraints. Medical education can provide a series of orientation maps, notably in the form of clinical practice guidelines and protocols. These are essential signposts for novices. In contrast, our participants, masters of the clinical craft with a “fund of experience in the culture” (Dreyfus 2014, 199), often alluded to gestalts, for example, the physician who recognized “the shape” of a pulmonary embolism. Thus, this mode of thinking is more than a form of reasoning—it is a way of being in the world. It is a form of understanding that flows from the physician’s attunement and an empathetic grasp of the numerous dimensions of health, illness, and clinical environments.

### Tacit dimensions of being and doing in clinical practice

Phronesis is not purely cognitive; rather, it is a know-how, a mode of grasping a situation that depends essentially on who one is (Gadamer 1971). The wise judgment, the good decision, and the right action are grounded in a tacit background of ingrained dispositions that are both practical and moral. In the findings, we used clinical habitus to designate the practical dispositions, or ‘know how’ of the clinician, whereas ‘ethos’ referred to the physician’s personal characteristics. This distinction is somewhat arbitrary, as dispositions are themselves manifestations of the clinician’s character. Indeed, ‘habitus’ is the Latin translation of the Greek ‘ethos’. However, we found the distinction useful to organize our results and for the purpose of the discussion.

Practical dispositions are capabilities that enable clinicians to grasp situations in a quasi-automatic mode and to perceive affordances for actions. In other words, habitus enables one to discern what is at stake and what is to be done. In our data, the ‘what’ went beyond strict biomedical or techno-scientific issues. To discern the ‘what’ of the situation, its crucial elements, the clinician may, for example, invoke a “magic bubble” (P-10), pushing back on intrusive aspects of the environment; or, rely on “street-smarts…. (to) get out of trouble” (P-9); or, parse out the situation in smaller “digestible bits” (P-3). Such dispositions seem to be, to a large extent, tacit and only partially conscious. Physicians frequently talked about “gut feelings” (P-6), “instinct” (P-7) or, “intuition” (P-7, P-8) as informing their clinical decisions. This aligns with literature that describes how physicians use reasoning that is automatic and that draws on experiential inductive reasoning—in addition to more analytic hypothetico-deductive modes of reasoning—to inform decision making (Croskerry 2009a, 2009b; Adler 2022). According to Hubert Dreyfus and Stuart Dreyfus, practitioners’ intuition is indicative of accumulated know how and tacit knowledge that an expert acquires by “dealing with, and seeing the outcome of, a large number of concrete situations” (Dreyfus 2014, 199).

Ethos refers to the virtues of the individual, in an Aristotelian sense (Collins 1999; Deslauriers 2002; Jimenez 2016), that is, character shaped by an education embedded within a set of shared norms, beliefs and values that is conducive to a good life. Our participants discussed several ‘clinical virtues’: humility, responsibility, a willingness to act, and being meticulous, available, and responsive. The good life in medicine entails good clinical practice: it may be precisely because clinicians have developed an appropriate ethos and clinical habitus that they are able to provide excellent care. In the material, ethos seems to be shaped by experiences, clinical role models, and values instilled across the life span, including parental influences and early childhood upbringing: “if I don’t do it, I feel bad about myself…I hear my mother’s voice in my head” (P-4). Such experiences appear to become integrated and embodied in the physician’s character and dispositions.

The connection between ethos and habitus is also manifest in the idea that medicine is more a vocation than a job. Vocation is grounded in the physician’s character and enacted in practical modes in the clinic: “there’s a certain passion and intensity that allows you to extend beyond the rules” (P-7). The participants’ embodied the values of medicine rooted in a clinical tradition and modelled by their teachers. The ethos is acquired through a process of socialization, in which the apprentice is willing to be transformed by the practice, and will, in turn, transform the practice of a clinician and teacher.

### Caring clinical responses

We have discussed that phronesis deals with practical situations and outlined their characteristics. We now wish to further describe the specific *clinical* nature of the situations the participants shared with us—situations that have the patient as a focal point. This was made visible by an array of *caring responses* described by the participants. Here, we refer to their dedication and concern for the patient and their emotional involvement. Participants spoke of their careful listening to the patient, thereby avoiding early interruption. They devised creative workarounds to circumvent systemic and institutional constraints and to deliver the quality of care they deemed appropriate and necessary. They demonstrated an intense dedication to their patients, with accounts that brimmed with tenderness and were often moving. Most expressed a profound regard for patients and their life experiences. A recurring notion was that of treating patients as if they were family members, which implied a deep personal commitment—as stated by one participant, “I have them and they have me” (P-9). This engaged and caring attitude is not consistently associated with phronesis in the literature. Notwithstanding, it is worth noting that Svenaeus (2022) argued that phronesis incorporates a feeling component and that this feeling component is empathy, where empathy is considered a kind of discernment — a way of seeing the world we share with others. Hubert Dreyfus and Stuart Dreyfus, for their part, have defended that phronesis is predicated on a capacity for a “caring response to the unique situation” (Dreyfus and Dreyfus 2014, 199). We suspect, indeed, that clinical phronesis is distinct from phronesis in other contexts because it includes such a caring response oriented toward the patient.

### Je-ne-sais-quoi

One finding in the material which we grappled to understand were statements with an elusive quality. Participants used expressions such as “hearing little voices” (P-10), following a “sixth sense” (P-5) or a“certain sense” (P-9), “feeling the wind” (P-6), and “praying” (P-4). Perhaps such statements can be related to two distinct but interconnected aspects of clinical practice. First, as we discuss above, phronesis involves a tacit know-how; this know-how is difficult to express in words and tends to be evoked using metaphors and approximations. Second, these statements speak to the impossibility of a complete mastery of the clinical situation, which is messy, heterogeneous, indeterminate, and dynamic. In contrast to the chess master, who is able to grasp the totality of the chess board, the expert clinician is working within contingent situations. In the ambiguities of practice, physicians are looking for ways to orient themselves, a path to follow, strategies to navigate the unpredictable, and resources to deal with inevitable limitations. This may account, in part, for their occasional references to hope, faith, and prayer.

## Conclusion

This empirical study has permitted us to develop detailed observations of how phronesis is manifest in (exemplary) physicians’ stories of clinical practice. We offer the term ‘clinical phronesis’ as a descriptor for the appearance and role of phronesis in daily medical practice. This term was previously alluded to in a perspective piece, written by Schultz and Carnevale (1996), on responsible caregiving. Their commentary was focused on ethics. They proposed clinical phronesis as a specific kind of virtue ethics. Our empirical study broadens the phenomenological understanding of the nature of phronesis within clinical settings. It also complements the contributions of scholars who have examined various features of clinical practice from the theoretical perspective of phenomenology (Baron 1990; Cooper 1994; Leder 1990; Svenaeus 2014).

The picture of clinical work that emerges is at odds with a portrayal of medicine that presents a detached physician, equipped with the knowledge of bioscience and trained in pre-defined competencies, who arrives at a diagnosis and selects therapies following algorithmic practice guidelines. We learned that exemplary clinical care is grounded in a particular ethos. A physician with a refined clinical habitus, shaped by accumulated experience, can grasp and make sense of a specific situation. Wise practice is pragmatic, intersubjective, and personal in being tailored to the individual as well as relational and caring in its enactment. The mode of reasoning, developed through years of practice, is hermeneutic and often tacit and intuitive.

The clinical situation comprises both patient and physician and is shaped by them jointly, against a backdrop of a myriad of contextual factors. These elements are encompassed within the clinical situation with the patient as the focal point of care. A physician with a fine grained and culturally engendered clinical habitus can grasp, elaborate, and organize a difficult and often messy clinical situation. Such a phronimos accompanies the patient through a thicket of choices in an environment laden with contingencies. It is our hope that this picture of ‘clinical phronesis’, grounded in the accounts of exceptional physicians, makes the concept more tangible and accessible and offers insight into how it may serve as a ‘guiding light’ for practice (Flaming 2001) and a ‘guiding logic’ for medical education (Kinghorn 2010).

## Footnotes

1 The interview transcripts are coded from P-1 to P-11 indicating the initial hour-long interviews. A follow up second interview transcript is coded FU P-1 to FU P-11.
